# The nature of malpractice claims related to nerve damage after dental implants insertion in Israel during 2005–2020: A descriptive study

**DOI:** 10.1111/cid.13163

**Published:** 2022-11-21

**Authors:** Amir Laviv, Roni Kolerman, Eitan Barnea, Nirit Tagger Green

**Affiliations:** ^1^ Department of Oral and Maxillofacial surgery, The Maurice and Gabriela Goldschleger School of Dental Medicine Sackler Faculty of Medicine, Tel Aviv University Tel‐Aviv Israel; ^2^ Department of Periodontology and Oral Implantology, The Maurice and Gabriela Goldschleger School of Dental Medicine Sackler Faculty of Medicine, Tel Aviv University Tel Aviv Israel; ^3^ Private Practice Tel‐Aviv Israel

**Keywords:** dental implants, lawsuit, malpractice, nerve injury

## Abstract

**Introduction:**

Dental implant placement is a routine practice in dentistry, with a possible uncommon risk of neurosensory injury. The present study analyzed all dental implant claims involving sensory nerve disturbances between 2005 and 2020 in Israel. The study was conducted to understand implant risk management better and improve the patient's safety.

**Materials and methods:**

All legal claims registered by Medical Consultant International (MCI) involving nerve damage claims between 2005 and 2020 were included in the study. The data included demographic details such as age, sex, event date, claim delivery date, and treatment settings. Information on the nerve damage included the damaged nerve, side of injury, and the number of implants performed in the same surgery.

**Results:**

There were 218 claims regarding nerve damage out of 1154 claims for dental implant therapy. The mean age for nerve damage claims was 54.1 ± 11 years.

There were more female than male claims (*p* = 0.02), with 87% of cases concerning damage to the inferior alveolar nerve (*p* < 0.0001), out of those molar areas being more frequently involved in nerve damage (64.3%, *p* < 0.0001).

The left side was 1.4 times more frequent than the right side (*p* = 0.043). The risk for nerve injury was 7.4 times higher when placing multiple implants compared to single dental implant (*p* < 0.0001).

**Conclusions:**

Clinicians should be aware that placement of multiple implants, left‐side implant placement, and patient gender may increase risk for a malpractice claim for neurosensory disturbances.

**What is known**

Legal proceedings involving dental professional liability claims have increased in recent years and are projecting on healthcare spending throughout the world. As the number of general dentists performing dental implants rises, complications and unwanted consequences are increasing, with possible major complication of sensory disturbances.

**What this study adds**

In order to successfully manage the possible neurosensory injury, it is essential to evaluate potentially general, intraoperative, and postoperative‐related risk factors. Such factors include the *gender* of the patient, the *jaw*, specific *site* and *side* of implantation, and the *number* of implants to be placed.

## 1 INTRODUCTION

Legal proceedings involving dental professional liability claims have increased in recent years and are projecting on healthcare spending throughout the world.^1^, ^2^, ^3^ As the number of general dentists performing dental implants rises, complications and unwanted consequences are increasing,^4^ with the possible major complication of sensory disturbances.^5^ Sensory damage can involve the peripheral branches of the trigeminal nerve; most expected the inferior alveolar nerve (IAN), the mental nerve, or the lingual nerve. Such injuries can lead to transient or permanent sensation loss or neuropathic pain, which can be challenging to treat.^6^

Possible causes of nerve injury include local anesthesia injection, direct nerve injury during flap incision and reflection, thermal injury‐induced osteitis (from insufficient irrigation during bone drilling), direct nerve contact, intrusion of bone debris in the mandibular canal during drilling, or implant‐mediated nerve compression (insufficient spacing between the implant apex and the mandibular canal) or cutting.^7^, ^8^ The damage that causes a neurosensory deficiency can occur during every step of the surgical procedure; that is, administration of local anesthesia, flap elevation, drilling, or placement of the dental implant.^4^ The prevalence of transitory nerve injuries is 0%–24%, while 0%–11% was reported for permanent damage following dental implant placement.^9^

Inferior alveolar nerve injury is a surgical complication that can interfere with the patient's quality of life, thus exposing the dentist or oral surgeon to a risk of medicolegal action.^10^ These injuries can negatively affect the patient's self‐image, quality of life, and psychological status that (in extreme cases) may include suicidal thoughts because of their pain.^11^

In Israel, dental insurance for almost 95% of dentists is performed by “Medical Consultant International” (MCI) company (Subsidiary of “Madanes” group). Therefore, the malpractice claims insurance data is valid, reliable, and available for litigation claims. This data is essential for the dental community to lower the chances of litigation and improve the patient's safety.^12^ However, there may be underestimation of legal claims, due to unknown number of patients who did not proceed with legal claims, and therefore were not documented anywhere. The present study aims to retrospectively analyze all dental implant claims delivered between 2005 and 2020 in Israel involving sensory nerve disturbances. The research was performed using a computerized database of MCI insurance company.

## 2 MATERIALS AND METHODS

The current study analyzed legal claims registered by MCI between January 1, 2005 and December 31, 2020. The Tel‐Aviv University local international review board (IRB) approved the research. Inclusion criteria included all claims related to dental implant insertion involving nerve damage present as part of the lawsuit.

The collected data was anonymous, comprising only identification claim number data to avoid duplication. Collected data included demographic details such as age, sex, date of the event, date of claim delivery, and treatment settings (private practice vs public clinic). Information on the nerve damage included the damaged nerve, side of injury, the expected implant that caused the damage, and the number of implants performed in the same surgery (one implant vs two or more).

### 2.1 Statistical analysis

SPSS software (IBM SPSS Statistics for Windows, version 25.0, IBM Corp.) was used for all statistical analyses. Categorical variables were analyzed using the Chi‐squared test, and continuous variables were analyzed using a *t* test. A *p*‐value of <0.05 was considered statistically significant.

### 2.2 Results

Between January 1, 2005 and December 31, 2020, there were 218 dental implants claims regarding nerve damage out of 1154 claims for dental implant therapy. The mean age for nerve damage claims was 54.1 ± 10.99 years. The time lag between the injury and the claim delivery was 3.04 ± 1.92 years.

Table 1 summarizes the demographic data analyzed. There were 1.4 more claims delivered by female patients (*p* = 0.02). The mandible was much more frequent than the maxilla, with 87% of cases involving damage to the IAN compared to other nerve injuries (lingual nerve or other nerve damage, *p* < 0.0001). Regarding the site‐ the molar area was 1.8 times more frequent in causing nerve damage (*p* < 0.0001).

**TABLE 1:** Demographic data frequencies in nerve damage cases

		Total (%)	*p*
Sex	Male	91 (41.7)	0.018
	Female	127 (58.3)	
Jaw	Maxilla	16 (7.5)	<0.0001
	Mandible	188 (87.9)	
	Maxilla and mandible	10 (4.7)	
Side of damage	Right	81 (41.3)	<0.0001
	Left	110 (56.1)	
	Bilateral	5 (2.6)	
Damaged nerve	Inferior alveolar	186 (86.9)	<0.0001
	Lingual	8 (3.7)	
	Other	20 (9.4)	
Implant area	Molar	83 (64.3)	<0.0001
	Premolar	43 (33.3)	
	Anterior teeth	3 (2.3)	
Implants installed	One implant	20 (11.8)	<0.0001
	Two or more implants	150 (88.2)	
Clinical type	Private practice	123 (68)	<0.0001
	Public practice	58 (32)	

The left side was 1.4 times more frequently damaged compared to the right side (*p* < 0.001). Analyzing the number of implants inserted in the same procedure—placing two or more implants were 7.4 times more frequent compared to single implant installation (*p* < 0.0001). Public practices were more frequently involved than private practices (68% compared to 32%, *p* < 0.0001).

There was no correlation between the gender to the side of damage or implant area (molar, premolar, and anterior), and neither between the implant site and the side of damage (left and right).

When comparing the molar area to the premolar area, the mean age was lower for nerve damage in the premolar area than in the molar area (51.58 ± 12.22 and 58.14 ± 10.71, respectively, *p* = 0.007).

## 3 DISCUSSION

Dental implant placement harbors an uncommon risk of neurosensory injury when the procedure is performed in the posterior mandible above the course of the inferior alveolar canal and mental foramen. In the present study, 18.9% of lawsuits in the case of dental implant therapy were due to nerve injury. The mean age of claimants was 54.1 years, similar to other studies, reporting claimants' age between 40 and 60 years.^13^

The incidence of dental implant‐related nerve injury out of the total dental implants claims in our study is lower than in some other studies.^3^, ^14^, ^15^ The studies reported altered sensation in the mandible following implant placement to be 37%, 29.8%, and 26%, respectively. However, the latter study^3^ published in 2002 reported the use of cone beam computerized tomography (CBCT) only in three out of 61(5%) reported cases.^5^ The importance of using CBCT was further evaluated by the same group from Israel in a more recent work from 2013. At that time point, 76% of the neurosensory deficiencies occurred when the surgeries were planned and performed based on panoramic or periapical radiographs only (without CT scans), whereas neurosensory damage occurred less frequent when surgeries were planned based on CT.^16^ A recent study from Finland concluded a fall in the rate of compensable malpractice cases concerning implant failure simultaneously with the use of CBCT radiographs.^15^ In a later study, Givol and colleagues reported that long‐term neurosensory changes occurred in 13% of patients.^16^ Our study relates to the years 2005–2020, hence the CBCT was used in most cases of dental implants in the posterior mandible, and therefore the neurosensory deficiency was lower.

Altered sensation after mandibular implant placement can result from trauma to any of the branches of the mandibular nerve, including the inferior alveolar, mental, and lingual nerves. The data revealed that the mental or IANs were damaged in most cases, but the lingual or maxillary nerve injuries were also evident at 3.7% (8 patients) and 9.4% (20 patients), respectively. These findings are in concordance with other studies of dental implant nerve damage that reported 32.2%–64.4% of IAN injury and 2.5%–28.8% of lingual nerve damage.^4^, ^13^ Many implant drills are slightly longer than their corresponding implant lengths for complete full implant placement at bone level. Implant drill length may vary and must be understood by the surgeon because the specified length may not reflect an additional millimeter, “y” dimension.^4^ It is recommended to examine mandibular nerve function before implant placement to determine whether there was a preexisting altered sensation.

Analyzing the number of implants inserted in the same procedure—there was a statistically significant difference between nerve injury in the installation of a single implant (20 cases, 11.8%) versus two or more implants (150 patients, 88.2%). Our results are in accordance with a previous study that confirmed that neurosensory deficiency occurred when more than one implant was placed in 79.4% of cases.^16^ This could be expected because multiple implant placements invariably involve different location drillings, more prolonged operation, a more extensive flap elevation, and more possible orientation difficulties.

The most significant host factors related to implant dentistry are the patients' age and gender. In our study, 58.35% of nerve injuries occurred in women compared to 41.7% in men (*p* = 0.02). It is well documented for all types of nerve injuries that females and increasing age are at higher risk of neurosensory deficits.^13^, ^17^ Over‐representation of women in these iatrogenic nerve injuries has been reported and discussed, with various explanations proposed. The greater tendency of women to seek dental treatment and to sue for malpractice are possible explanations. Still, there may be a biological reason for this phenomenon widely seen in other trigeminal nerve injuries and injuries of different etiology. Greater neurogenic vulnerability remains to be elucidated.^18^

The age was statistically significant, with a lower mean age for nerve damage in the premolar area than nerve damage in the molar area (51.6 and 58.1, respectively). The higher percentage of injury to the IAN in the molar area is more prevalent in older age.

Injury in the area of the premolars might be caused due to an injury to the mental nerve/foramen. The foramen is usually located by the apex of the second mandibular premolar or between the apices of the premolars. Minor variations may be race related. After extraction of teeth and resorption of alveolar bone, the mental foramen is closer to the alveolar crest. In extreme cases, the mental foramen and mandibular canal can be adjacent to the crest of the alveolar ridge. The anterior loop, an extension of the IAN, is anterior to the mental foramen before exiting the canal.^19^ Injury in the premolar region is mainly related to mental foramen injury. The average density and thickness of the bone surrounding the mandibular canal are insufficient to resist the implant drill.^20^ Cancellous bone is more abundant in the posterior mandible and has larger intra‐trabecular spaces but is less dense than in the anterior mandible. The decreased bone density might explain the higher injury rate in older ages.^4^

An innovative finding is the significantly higher rate of nerve damage on the left side compared to the right side: 110 injured nerves (58.3%) compared to 81 injured nerves (41.3%) on the right side (*p* < 0.0001). Currently, no studies deal with the significant difference between implant dentistry's right and left sides. The only study comparing sides of the jaws compared the efficacy of Coronally Advance Flap for localized gingival recessions. They revealed that complete root coverage (CRC) in teeth on the right side of the arches obtained a higher CRC than teeth on the left side (odds ratio 1.60, *p* < 0.05).^21^ As 70%–90% of the general population are right‐handed, a possible explanation for the higher number of left side nerve damage can be due to more challenging orientation to the surgeon, sitting on the right side of the patient and drilling on the left side, which can cause more inaccuracy during free hand drilling of dental implants.

### 3.1 Strengths and limitations

The MCI database used in this study covers the entire country, as almost 95% of the dental practitioners in Israel were professionally insured by this company during the 16‐year study period. This is a significant strength of our cohort. Another strength is the relatively high number of nerve injuries observed over a long period of 16 years.

However, the study has several limitations. First, the relevance of the results on the subgroups divided by gender and treatment setting (private vs public) is limited, as the division of the total number of patients undergoing implant treatment according to these subgroups is unknown. For example, although most claimants in our study were woman, we cannot conclude that it is riskier to treat a woman patient from a malpractice perspective. Instead, it may reflect that women are the majority of treated patients. Finally, due to the insurance company's objection, data on the compensation payments were not included.

## 4 CONCLUSIONS

The number of legal claims for implant therapy in Israel is increasing. Implant therapy is an elective procedure, and as such, the dentist must use every required diagnostic tool (such as CBCT) and use preventive measures such as keeping a safe distance from the nerve. As the inferior alveolar injury was most common, it is essential to evaluate potentially general factors such as the jaw, specific site and side of implantation, and the number of implants to be placed.

## AUTHOR CONTRIBUTIONS

Amir Laviv: Design, data collection and analysis, statistics, article writing. Roni Kolerman: Concept, article revision and approval. Eitan Barnea: Concept, article revision and approval. Nirit Tagger Green: Design, data collection and analysis, article writing.

## FUNDING INFORMATION

The author(s) received no financial support for the research, authorship, and/or publication of this article.

## CONFLICTS OF INTEREST

All authors declare no conflict of interest

## Data Availability

The data that support the findings of this study are available on request from the corresponding author. The data are not publicly available due to privacy or ethical restrictions.
